# Gene Discovery in the Threatened Elkhorn Coral: 454 Sequencing of the *Acropora palmata* Transcriptome

**DOI:** 10.1371/journal.pone.0028634

**Published:** 2011-12-28

**Authors:** Nicholas R. Polato, J. Cristobal Vera, Iliana B. Baums

**Affiliations:** Department of Biology, The Pennsylvania State University, University Park, Pennsylvania, United States of America; King Abdullah University of Science and Technology, Saudi Arabia

## Abstract

**Background:**

Cnidarians, including corals and anemones, offer unique insights into metazoan evolution because they harbor genetic similarities with vertebrates beyond that found in model invertebrates and retain genes known only from non-metazoans. Cataloging genes expressed in *Acropora palmata*, a foundation-species of reefs in the Caribbean and western Atlantic, will advance our understanding of the genetic basis of ecologically important traits in corals and comes at a time when sequencing efforts in other cnidarians allow for multi-species comparisons.

**Results:**

A cDNA library from a sample enriched for symbiont free larval tissue was sequenced on the 454 GS-FLX platform. Over 960,000 reads were obtained and assembled into 42,630 contigs. Annotation data was acquired for 57% of the assembled sequences. Analysis of the assembled sequences indicated that 83–100% of all *A. palmata* transcripts were tagged, and provided a rough estimate of the total number genes expressed in our samples (∼18,000–20,000). The coral annotation data contained many of the same molecular components as in the Bilateria, particularly in pathways associated with oxidative stress and DNA damage repair, and provided evidence that homologs of p53, a key player in DNA repair pathways, has experienced selection along the branch separating Cnidaria and Bilateria. Transcriptome wide screens of paralog groups and transition/transversion ratios highlighted genes including: green fluorescent proteins, carbonic anhydrase, and oxidative stress proteins; and functional groups involved in protein and nucleic acid metabolism, and the formation of structural molecules. These results provide a starting point for study of adaptive evolution in corals.

**Conclusions:**

Currently available transcriptome data now make comparative studies of the mechanisms underlying coral's evolutionary success possible. Here we identified candidate genes that enable corals to maintain genomic integrity despite considerable exposure to genotoxic stress over long life spans, and showed conservation of important physiological pathways between corals and bilaterians.

## Introduction

Cnidarians are valuable for understanding the evolution of metazoan genomes because they comprise a sister group to the Bilateria. From a developmental perspective, comparisons among these groups have yielded much information regarding the evolution of body plans in complex multicellular animals [1]. The two cnidarian genomes sequenced to date (*Hydra magnipapillata and Nematostella vectensis*) have revealed that basal metazoans retain genes known only from non-metazoans [2], and possess genetic pathways found in vertebrates but lacking in worm and fly models [3].

Corals in particular offer insights into important biological processes (including calcification and symbiosis) that are responsible for the formation of reefs worldwide [4]. These processes contribute to the fitness of coral genets. Successful genets can live to be hundreds of years old [5], [6], and because generation times for *A. palmata* are short by comparison (on the scale of 2 to 5 years [7]), a single successful genet can contribute an enormous number of offspring to future generations over its lifetime. Thus, chronic exposure to high levels of UV irradiation and radical oxygen species (ROS) produced as a byproduct of symbiont photosynthesis, is likely to have driven the evolution of powerful genoprotective mechanisms and DNA repair pathways in corals [8].

Progress in sequencing technology, chemistry, and bioinformatics now enable the sequencing and *de novo* assembly of whole transcriptomes from non-model organisms [9], [10], [11]. Such genomic resources are needed for species of conservation concern because they allow researchers to target functional variation in wild populations [12], [13], [14], track adaptive responses to environmental stress through space and time [15], and inform breeding programs and reintroduction efforts [16].

Despite a population reduction of >80% throughout its range in recent decades [17], the Elkhorn Coral (*Acropora palmata*) remains an ecologically important community member [18], [19], providing the three dimensional structure of many Caribbean reefs and a substantial contribution to reef primary productivity [18], [19]. Further, *A. palmata* is one of only a few Caribbean species for which population genetic data is available [20], [21], [22]. Microsatellite markers have revealed patterns of population differentiation and the contribution of asexual reproduction throughout *A. palmata's* range [23], [24] and as such, a baseline for genetic and genotypic diversity within this species exists.

Transcriptome and EST data are now available from several coral species including *Acropora millepora, Acropora hyacinthus, Montastraea faveolata, Pocillopora damicornis, and Porites astreoides* [9], [25], [26], [27], [28, Matz and Traylor–Knowles pers. comm.] enabling comparative genomics within the scleractinia [28], [29]. Likewise the genomes of two cnidarians, *Nematostella vectensis* and *Hydra magnipapillata* were recently completed [30], [31] permitting evolutionary analysis within the phylum.

To develop genome scale tools for *A. palmata* we present here its transcriptome sequenced with 454 GS-FLX technology, and survey the assembled and annotated sequences for genetic markers, and functional enrichment of important biological processes (with a particular focus on stress response and DNA repair pathways). The genetic resources presented here are a major advance for studies of this species and will promote the study of adaptive trait variation in wild populations of *A. palmata* as well as comparative genomics among basal metazoans.

## Results

### Biological material

RNA was acquired from genetically diverse larvae collected over a broad geographic range previously identified as comprising two divergent populations [23]. Larvae were exposed to three temperature and two CO_2_ treatments, and sampled over the course of development from fertilization to the planula stage. A small amount of adult tissue was also included to represent adult transcripts. We thus expected to find developmental and stress related genes derived from the larval temperature and CO_2_ treatments, as well as genes related to symbiosis and calcification from the adult tissue.

### Sequencing and Assembly

**Figure 1:**
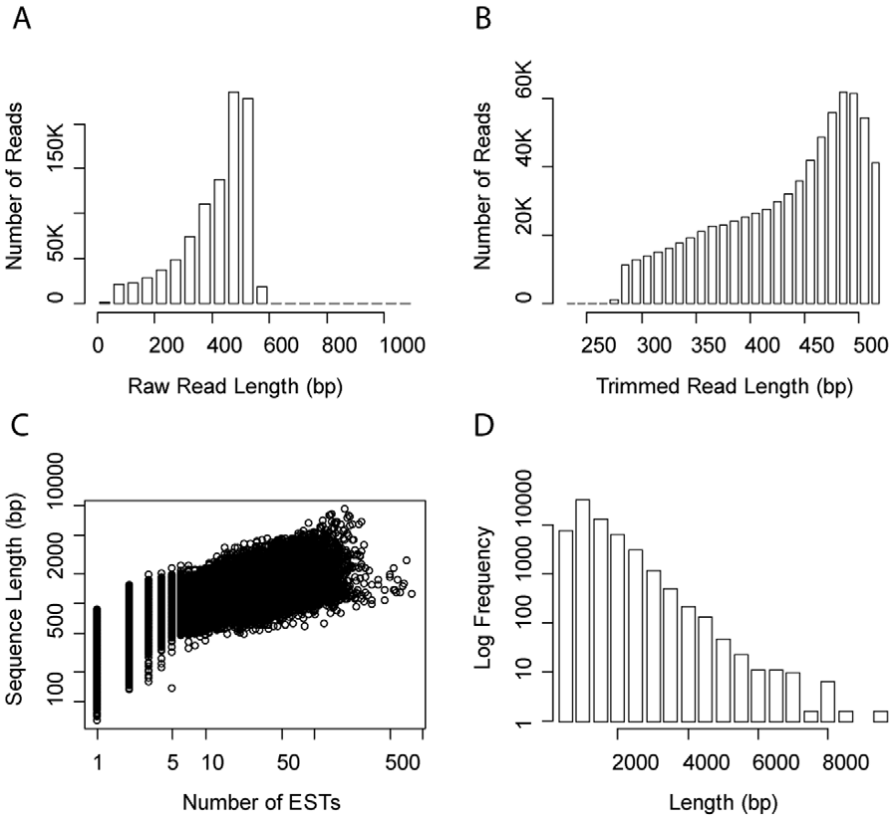
Summary of sequencing read length distributions from the *A. palmata* transcriptome pre and post assembly. A) Unassembled raw sequencing read lengths (in basepairs, bp) prior to trimming. B) Size distribution of unassembled sequences (in bp) following quality trimming. C) Plot of the relationship between the length of assembled contigs (in bp) and the depth of coverage in terms of the number of raw ESTs they include (note log-log axes). D) Frequency histogram of contig lengths (in bp).

One plate of a normalized cDNA library generated from a pooled *A. palmata* sample following the library preparation methods of *Meyer et al.* [9] was sequenced on the 454 GS-FLX platform using Titanium chemistry. This run yielded 964,519 raw reads with an average length of 398 bp (σ = 118), totaling of 384 Mb (Fig. 1A; Table 1). After trimming for size, quality, and primer sequence 741,271 reads remained averaging 432 bp (σ = 64) in length and totaling 320 Mb. The majority of trimmed reads (98%) were over 400 bp in length (Fig. 1B) contributing to the high quality of the assembly.

**Table 1:** Summary of sequencing and assembly of the *Acropora palmata* transcriptome.

	N sequences	Totallength [Mb]	Avg. length(sd) [bp]
raw reads	964,519	384	398 (118)
trimmed reads	741,271	320	432 (64)
contigs	42,630	44	1030 (623)
singletons	45,390	20	439 (94)
total	88,020	64	

Trimmed reads were assembled with a set of publicly available *A. palmata* ESTs (n = 36,236; SymBioSys database: sequoia.ucmerced.edu/SymBioSys/). Assembly yielded 42,630 contigs averaging 1,030 bp long (σ = 623). Contig sizes ranged from 132 to 9,066 bp. The mean depth of coverage was 5.6 sequences (Fig. 1C). The size distribution of contig lengths showed an abundance of large contigs resulting from the long read lengths (Fig. 1D). Over 88% (37,819) of the contigs were greater than 500 bp long, and over 38% (16,274) were greater than 1,000 bp. After assembly, 45,390 singletons remained that could not be incorporated into any contig. Due to their long average length (439 bp; σ = 94), and the fact that a substantial proportion of singletons are likely to represent low abundance transcripts [9], these sequences were included with the contig sequences in blast searches [32] for annotation information.

### Transcriptome Completeness

Estimation of transcriptome completeness based on two different methods suggest that our single sequencing run tagged between 83–100% of the genes expressed in *A. palmata*. A blast query of the assembled *A. palmata* sequences against a set of 119 orthologs conserved across metazoans and found to be single copy in cnidarians (see methods), showed high quality hits (e-value ≤2×10^−38^, bitscore ≥130) to all 119 genes. Comparison of the distributions of coverage depth between all *A. palmata* transcripts and those with hits to the 119 single copy orthologs showed that the mean depth of coverage for contigs with hits to these 119 genes (9.7) is greater than that for contigs in general (5.6). This analysis indicated that depth of coverage was high even for single copy genes and that the set of 119 orthologs can serve as a representative sample of the *A. palmata* transcriptome.

Secondly, by comparing our assembled data to the published *N. vectensis* transcriptome we found hits to 83% of all *N. vectensis* transcripts. The lengths of the *A. palmata* sequences were generally greater than those of *N. vectensis*, suggesting that full length transcripts were obtained for the majority of the *A. palmata* genes (Information S1). The distribution of log length ratios was shifted to the right of zero due to the fact that *A. palmata* sequences were longer on average than the corresponding *N. vectensis* sequence. The modal value of the length ratio distribution (106%) reflected the longer average lengths of the *A. palmata* transcripts, and taken together with the high proportion of hits to the *N. vectensis* transcriptome data indicated very high coverage of the *A. palmata* transcriptome.

### Annotation and Transcriptome Size

All contigs and singletons (n = 88,020 sequences) were used to search against the UniProt protein database. Searches of both Swiss-Prot and TrEMBL resulted in hits for 50,118 (57%) of the queried sequences, of which 32,114 (36%) represented unique subject names. 31,888 of these hits corresponded to Gene Ontology (GO) [33] annotations representing 109,664 terms (4,617 unique). Of the GO terms identified, 46% were molecular functions, 32% biological processes, and 22% cellular components.

To estimate annotation efficiency, the description terms associated with each of the 119 single copy genes were compared to those in the *A. palmata* dataset (derived from UniProt). A clear match was found for 88% of the 119 annotation descriptions. An additional 9% of matching *A. palmata* transcripts were described with uninformative species specific codes from UniProt, but functional matches were easily found by looking up the codes in the NCBI UniGene database. Only three of the 119 genes did not match with the *A. palmata* annotation data, and in all three cases the *A. palmata* annotation was derived from blast hits to the Florida lancelet or the green puffer, species with limited annotation data. Overall this suggests high (97%) annotation efficiency.

Assuming that gene duplication is not excessive (a reasonable expectation based on the results of the paralog analysis below), the number of assembled sequences matching to known single copy genes can give an estimate of the level of residual gene fragmentation (either from sequencing gaps or splice variants), and/or assembly difficulty. blast results from the 119 orthologs were used to examine the average number of hits to each putatively singe copy gene. Results of this test showed that 63% of the 119 single copy genes had multiple sequence matches (range: 1–19; mean = 2.2; σ = 1.95; Information S1) in the *A. palmata* data, suggesting a moderate amount of fragmentation in our data, with some outliers that may be related to gene size and/or species specific duplications.

The degree of fragmentation in our assembly was also used to generate a rough estimate of the total number of transcripts expressed in the *A. palmata* sample. The mean value of 2.2 contigs per transcript was used as an estimate of the redundancy in our dataset. This yielded an estimate of ∼19,377 genes (42,630 contigs ÷2.2) expressed in our *A. palmata* sample. Finally, based on the blast results with *N. vectensis* we found that 43,036 of the *A. palmtata* sequences matched to a non-redundant set of 17,988 proteins from *N. vectensis*. Thus we predict that the number of transcripts expressed in *A. palmata* is in the range of ∼18,000–20,000 genes.

### Taxonomic Annotation

The majority of taxonomic associations of the annotated transcripts matched to metazoan invertebrates and vertebrates (44% and 48% respectively). A small proportion of sequences matched to bacteria (3%), protists (2%), plants and algae (2%). All other groups including fungi, archaea and viruses accounted for 1% or less of matches. Stony coral (Scleractinian) sequences accounted for only 1% of the annotated sequences primarily due to a paucity of annotated coral sequences present in current protein databases.

Because blast annotation associates a given sequence with its closest match in the database, results are limited to previously identified genes. Thus, the vast majority of annotated transcripts in this dataset showed matches to the Starlet Sea Anemone, *Nematostella vectensis* (32,465 using best hit criterion), the most closely related organism with a sequenced genome [31]. Similarly, an abundance of cepheolochordate blast hits reflected the sequencing efforts on the Florida Lancet, *Branchiostoma floridae* [34]. Because annotation information is limited for both of these genomes we considered the next most informative blast hits in our annotation database when the best sequence match lacked any functionally relevant data. Viewed this way the number of *A. palmata* sequences with best matches to *N. vectensis* was reduced from 36% to 12%. Thus, despite slightly higher sequence similarity of hits to *N. vectensis*, the search for useful functional annotations was greatly improved by exploring more distantly related hits.

### Functional Annotation

**Figure 2:**
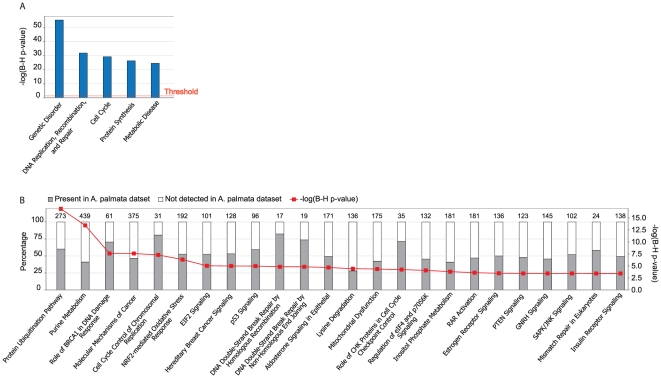
Enrichment analysis highlighted well annotated pathways that are of interest in heat stressed embryos. A) The five most highly enriched functional categories in the *A. palmata* dataset (the orange line represents the threshold for significance at p<0.05 with FDR adjustment for multiple testing. B) The top canonical pathways from the IPA library of pathways that were most significant to the dataset. The left Y axis shows the percentage of proteins in the pathway that were identified in the *A. palmata* data (grey bars, note that this is independent of expression levels). The right Y axis shows the corrected –log (p-values) for Fisher's exact test of the probability that the association between the pathway and the data is explained by chance.

To guide functional interpretation of *A. palmata* transcripts, we placed *A. palmata* homologs in the context of known biological pathways using ipa software (Ingenuity Systems; www.ingenuity.com). Information in the ipa database reflects the level of interest certain pathways have received. Thus, comparison of *A. palmata* transcripts to the IPA database identified well annotated metazoan pathways for which a high proportion of pathway components were present in *A. palmata*. These pathways were then used as guides for further analysis. Note that this approach did not make inferences about expression levels of transcripts. Among the most highly represented pathways were many that are expected to be important to heat stressed embryos and who are thus of interest to the current study including protein ubiquitination, cell cycle control, NRF2 mediated oxidative stress response, p53 signaling and DNA double stranded break repair (Fig. 2).

#### DNA damage and oxidative stress

**Figure 3:**
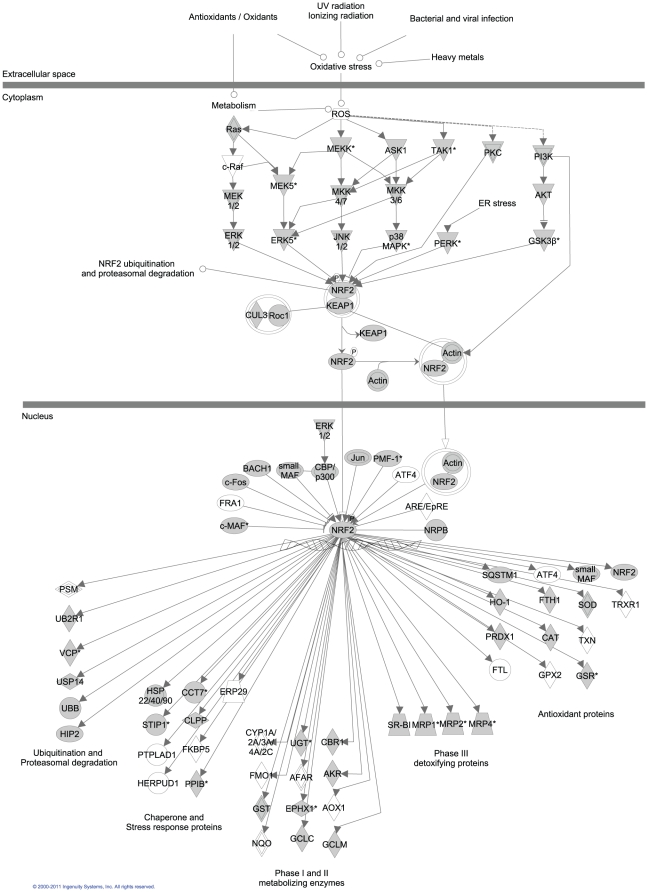
Homologs involved in canonical oxidative stress response pathways identified in the *A. palmata* transcriptome. Filled symbols indicate proteins with homologs that were detected in the *A. palmata* dataset. Unfilled symbols indicate proteins that were not detected in our data, but are present in model vertebrate pathways.

**Figure 4:**
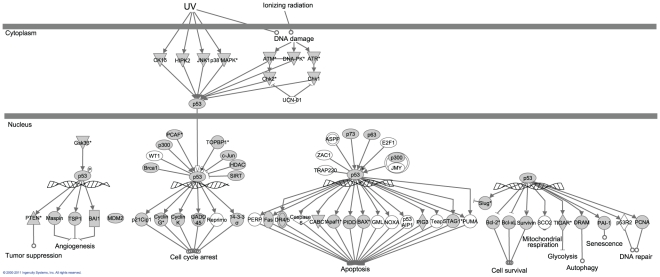
Genes involved in the various p53 mediated responses to DNA damage. Specific pathway details are likely to differ somewhat in the Cnidaria, but the presence of these components in *A. palmata* suggested the capacity for similar responses in corals. Filled symbols indicate proteins with homologs that were detected in the *A. palmata* dataset. Unfilled symbols indicate proteins that were not detected in our data, but are present in model vertebrate pathways.

Genes involved in the response to DNA damage and oxidative stress (Figs 3 & 4) included homologs of the transcription factors HIF1α and NRF2 responsible for promoting expression of numerous proteins with oxidoreductase activity [35], [36]. Additionally, a homolog of KEAP1, the primary regulator of NRF2, [37] was identified along with other transcripts involved in the detoxification, repair and removal of damaged proteins. A multitude of heat stress response genes were found, including 13 HSP variants, numerous HSP binding proteins, and associated transcription factors.

#### Selection in p53 family genes

**Figure 5:**
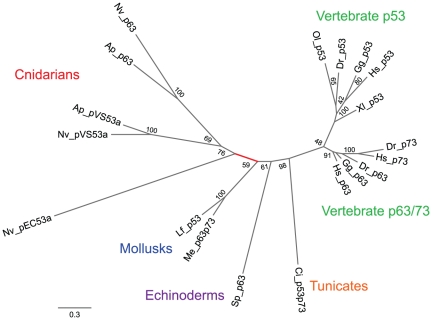
p53 gene family tree. Tests were performed to detect evidence of natural selection along the branch leading to the Cnidaria with the program PAML (highlighted in red). The sequences in the tree include a subset of those from Rutkowski (2010). The tree was generated in garli using the TIM2+G model.

Next, we placed genes involved in the response to DNA damage and oxidative (and irradiant) stress in a phylogenetic context to test for signatures of natural selection. 498 bp of the conserved DNA binding domain from homologs of two *N. vectensis* p53 family members (pVS53a, and p63), were chosen to test for positive selection with the program paml [38]. A third homolog (pEC53a) was also identified but the sequence did not include the conserved DNA binding domain and could not be included in the alignment. Results of a likelihood ratio test (p = 0.02; 2ΔlnL = 11.4, d.f. = 4,) comparing a “nearly neutral” model with two site classes (dN/dS = 0 and dN/dS = 1), and a “positive selection” model that included a third class with a dN/dS ratio >1, supported the hypothesis that positive selection has occurred on the branch of the tree separating cnidarian and bilaterian p53 family members (Fig. 5; Information S2).

#### Calcification

Calcification is crucial for coral growth and survival, and numerous genes involved in coral calcification were found in the *A. palmata* transcriptome. These included 68 transmembrane ion transporters, including 10 sodium driven bicarbonate exchangers which may play an important role in supplying bicarbonate ions to the reaction site of calcification. Another enzyme, carbonic anhydrase found in the coral calicodermis (a cell layer at the interface of the polyp and skeleton that secretes organic molecules to promote biomineralization) [39], catalyzes the hydration of carbon dioxide leading to hydrogen and bicarbonate ions, which in turn react with calcium ions to form the calcium carbonate skeleton. Twenty-four hits to carbonic anhydrase enzymes were found in our dataset including a homolog of α-CA recently cloned from *Stylophora pistillata* [40].

### Paralog Group Analysis

**Figure 6:**
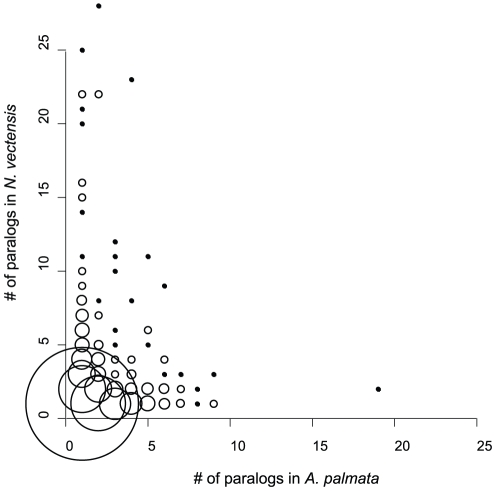
Distribution of paralog groups identified between *A. palmata* and *N. vectensis* by inparanoid. Plotting on a gene by gene basis showed that genes with large numbers of paralogs (>5) differed between species. Groups with a single member from each species were most common, and Groups with >5 members each accounted for less than 0.5%. Only a single group with >9 members was found in *A. palmata*. Circle size is proportional to the number of groups of a given size class.

Identification of orthologous and paralogous transcripts in a species pair provides information regarding gene duplication prior to and following speciation, and may point out gene families of particular importance to certain species. Between *N. vectensis* and *A. palmata*, 7,754 ortholog pairs were detected, with 9,604 in-paralogs in *N. vectensis* and 9,365 in-paralogs in *A. palmata*. The size distribution of paralog groups showed that the number of groups detected declines with increasing group size, and groups of 5 or more were rare (Fig. 6). Functional annotation of genes with multiple paralogs (group size >5) in *A. palmata* revealed a number of transcripts of known importance to corals, including green fluorescent proteins, carbonic anhydrase, and the oxidative stress response gene ferritin (Table 2). Interestingly among the largest groups identified were homologs of the immunoglobulin superfamily proteins in the IgLON family (Immunoglobulin superfamily containing LAMP, OBCAM, and neurotrimin), and several tumor necrosis factor receptor-associated factors (TRAFs).

**Table 2:** Paralog group size and identity for paralog groups identified in *Acropora palmata* as compared to the *Nematostella vectensis* proteome.

# of Paralogs	Descripton	Gene Function
19	IgLON family member	Immunoglobulin domain; Cell Adhesion Molecule
9	TNF receptor-associated factor 2/6	Signal transduction; Apoptosis
8	Egg protein	
7	Ferritin	Iron; Ironstorage; Metal-binding
7	Egg protein	
6	Triglyceride lipase	Hydrolase
6	GPI-linked carbonic anhydrase	Lyase
6	Chromoprotein	3D-structure; Chromophore; Luminescence; Photoprotein
6	Neuronal pentraxin-1	Calcium; Metal-binding
6	G-protein coupled receptor	Receptor
6	Myosin VIIA	ATP-binding; Motor protein; Myosin; Nucleotide-binding
6	Cupin family protein	
6	Dopamine beta-monooxygenase-like protein	Monooxygenase
6	TNF receptor-associated factor 3/6	Metal-binding; Signal transduction; Apoptosis
6	Egg protein	EGF-like domain
6	SH3 and PX domain- containing protein 2A/B	Cell projection
5	TNF receptor-associated factor 6	Metal-binding; Ubl conjugation; Signaling; Apoptosis
5	mab-21-like 1 (MAB21L1)	Cell proliferation
5	Sulfotransferase	Transferase
5	EGF like domain containing protein	
5	LPS-induced TNF-alpha factor	Transcription regulation; Apoptosis
5	Fucose binding lectin	Lectin
5	Putative SAM-dependent methyltransferase	Methyltransferase
5	CCAAT/Enhancer binding protein beta	DNA-binding
5	Skeletrophin, putative	Hydrolase; Metal-binding
5	Rho guanine nucleotide exchange factor 7	Guanine-nucleotide releasing factor; Phosphoprotein
5	Chromobox protein homolog 7	Chromatin regulator; Transcription regulation
5	Neuroblast differentiation associated protein	

### Microsatellite and SNP discovery

**Figure 7:**
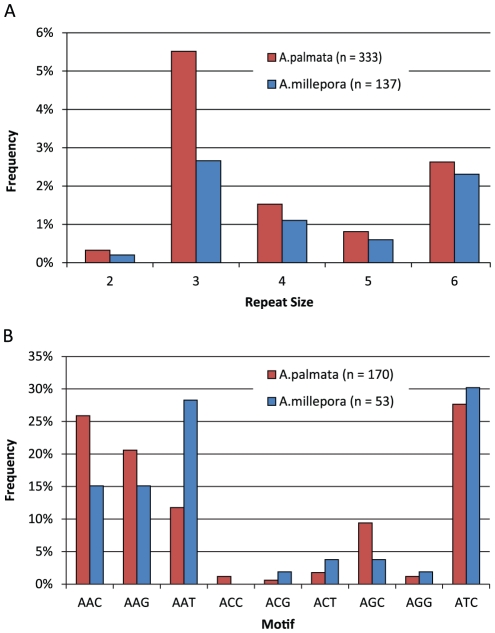
Microsatellite repeat distributions show similar patterns in *A. palmata* (red) and *A. millepora* (blue). A) Microsatellite frequency distributions showed an abundance of tri and hexanucleotide repeats in both species. B) Trinucleotide motifs were dominated by ATC repeats in both species, followed by varying proportions of AAN type repeats. GC rich repeats were rare, and no CCG repeats were found.

A total of 333 microsatellites (defined as repeats with motifs between 2 and 6 bp) were found, with a large majority consisting of trinucleotide repeats (n = 170; Fig. 7). Within the trinucleotides, AAN type repeats were most abundant, with the highest number being AAC repeats, followed by AAG, and AAT (44, 35, and 20 respectively).

**Figure 8:**
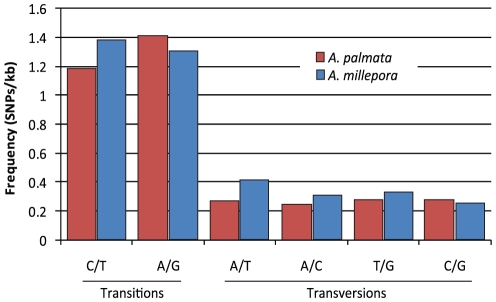
Frequency of various single nucleotide polymorphism (SNP) types in *A. palmata* (red) and *A. millepora* (blue). Frequencies are given per 1000 bp. Transitions are in red and transversions in blue. The overall frequency of SNPs in *A. palmata* was 1 per 272 bp.

**Figure 9:**
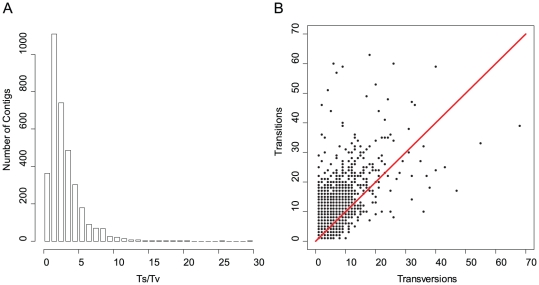
Variation in transition to transversion ratios (Ts/Tv) among contigs. A) The histogram of Ts/Tv ratios showed a number of contigs with ratios <1. B) Plotting the number of transitions (Ts) against the number of transversions (Tv) showed genes that have appreciable numbers of both SNP types. The diagonal line represents a ratio of 1, points below the line have a ratio <1 that may indicate a history of positive selection.

A total of 72,605 candidate SNPs were identified from 13,803 contigs spanning 19.8 Mb of sequence. The overall SNP frequency was 1 per 272 bp, with 71% transitions (Ts) and 29% transversions (Tv). Frequencies of different transitions types were similar, as were frequencies of different transversion types (Fig. 8). The transcriptome wide Ts/Tv ratio was 2.4, and while most contigs (55%; n = 3,823) had ratios of 2 or greater, many contigs had ratios that were substantially higher or lower (Fig. 9). Contigs with especially high and low ratios were analyzed to identify possible genes under selection and associated functional groups (Table 3). These contigs included genes involved in TGF-β, Hedgehog, and WNT signaling pathways; DNA and protein repair; lipid, protein and energy metabolism; HSP binding; oxidation-reduction; and regulation of transcription (Information S3).

**Table 3:** Significantly enriched GO terms associated with transcripts showing especially low (<1) Ts/Tv ratios (Upper) and high (>5) Ts/Tv ratios (Lower).

Ts/Tv Class	Primary GO Level	n (K<1)	n (total)	% (K<1)	% (total)	p value	GO ID	GO term
Low (K<1)	Biological Process:	64	681	7.6	9.8	0.05	GO:0006807	nitrogen compound metabolic process
		56	609	6.7	8.7	0.04	GO:0006139	nucleic acid metabolic process
		124	1296	14.8	18.6	0.01	GO:0044237	cellular metabolic process
		69	774	8.2	11.1	0.01	GO:0043473	pigmentation
		69	748	8.2	10.7	0.03	GO:0050794	regulation of cellular process
		69	773	8.2	11.1	0.01	GO:0050789	regulation of biological process
	Cellular Component:	211	2124	25.1	30.4	0.00	GO:0044464	cell part
		127	1423	15.1	20.4	0.00	GO:0044424	intracellular part
		81	963	9.7	13.8	0.00	GO:0043231	intracellular membrane-bounded organelle
		10	177	1.2	2.5	0.02	GO:0044428	nuclear part
		74	820	8.8	11.7	0.01	GO:0005737	cytoplasm
	Molecular Function:	250	2511	29.8	36	0.00	GO:0005488	binding
		75	806	8.9	11.5	0.02	GO:0005515	protein binding

Ts/Tv Class	Primary GO Level	n (K>5)	n (total)	% (K>5)	% (total)	p value	GO ID	GO term
High (K>5)	Biological Process:	72	649	11.8	9.3	0.05	GO:0009058	biosynthetic process
		61	514	10	7.4	0.02	GO:0010467	gene expression
		108	1010	17.6	14.5	0.03	GO:0044260	cellular macromolecule metabolic process
		134	1296	21.9	18.6	0.04	GO:0044237	cellular metabolic process
		3	106	0.5	1.5	0.04	GO:0044262	cellular carbohydrate metabolic process
		96	875	15.7	12.5	0.03	GO:0019538	protein metabolic process
		37	301	6	4.3	0.05	GO:0080090	regulation of primary metabolic process
	Cellular Component:	152	1423	24.8	20.4	0.01	GO:0044424	intracellular part
		123	1178	20.1	16.9	0.04	GO:0043226	organelle
		65	521	10.6	7.5	0.01	GO:0044444	cytoplasmic part
	Molecular Function:	17	110	2.8	1.6	0.03	GO:0005198	structural molecule activity
		88	806	14.4	11.5	0.04	GO:0005515	protein binding

Low Ts/Tv ratios may be a rough signal of positive selection, while elevated ratios could indicate purifying selection.

To date, 18 SNPs from 6 contigs have been targeted for amplification using PCR, and the products were sequenced on an ABI3700 fragment analyzer. Of these 18 SNPs, 8 were observed in the sample population (success rate 44%), which consisted of 11 individuals from Florida, 8 from Curacao, and 7 from Puerto Rico. The SNPs detected were in genes for NFkB, melanopsin, Cyano Fluorescent Protein, NRF2, galaxin, and LITAF (Information S4). Ongoing work will develop a panel of SNPs for surveys of functional variation across the species' range.

## Discussion

Coral species are the foundation of reefs because of their association with intra-cellular photosymbionts and ability to calcify. Comparative functional genomic analyses are now possible to elucidate the mechanisms underlying coral's evolutionary success. The *Acropora palmata* transcriptome presented here joins transcriptomes and EST data available for several other scleractinian corals, including that of the Pacific congener *A. millepora* (Matz and Traylor-Knowles pers. comm.) [9], [25], [26], [27], [28]. The distribution of transcript sizes observed in *A. palmata* (mean = 1,030 bp; median = 830 bp; σ = 623 bp) was comparable to that of *N. vectensis* (mean = 1,091 bp; median = 806 bp; σ = 1073 bp), and the estimated number of genes expressed in this species (∼18,000–20,000) was within the range estimated for *A. millepora* (∼11,000) and *N. vectensis* (27,273).

The high quality and comprehensiveness of the assembled data enabled functional analysis of pathways shared among metazoans as well as those of particular importance to the Cnidaria. *A. palmata* possesses many of the molecular components present in the more complex Bilateria (particularly in pathways associated with oxidative stress and DNA damage repair) and homologs of p53, a key player in several DNA repair pathways, showed evidence of natural selection along the lineage separating Cnidaria and Bilateria.

### Taxonomic Annotation

Xenobiotic sequences from intracellular parasites are commonly found in transcriptome data of wild species [10]. Many of the non-coral sequences identified here may represent symbiotic or pathogenic organisms associated with *A. palmata*. Indeed, approximately half of the sequences with blast hits in the ‘protists’ category (555) were associated with the superphylum which includes symbiotic zooxanthellae.

Hits to sequences from scleractinians were rare because of the small number of coral genes in current databases (0.01% of total). As such, the genes identified are likely to be highly conserved among the scleractinians, with remaining unannotated transcripts (43% of all assembled sequences) representing a combination of coral specific genes [41] and genes that are too divergent from those in existing databases to identify using blast with reasonable cutoff parameters.

### Functional Annotation

#### Oxidative Stress

Corals live in shallow, warm waters and house intra-cellular photosymbionts resulting in exposure to high levels of UV radiation and oxidative stress [8]. Excessive oxidative stress and UV exposure can damage nucleic acids [42], [43] and these conditions may have selected for powerful and efficient DNA repair mechanisms in corals. Homologs of a majority of genes involved in the canonical pathway for oxidative stress response (as identified in model organisms) were present in the *A. palmata* transcriptome (Fig. 3) suggesting that corals may deal with high oxidative stress loads using many of the same biochemical mechanisms as the Bilateria.

#### DNA damage repair

Our data includes a coral homolog of the mutS gene which is involved in mismatch repair and is thought to be responsible for the low mtDNA mutation rate observed among cnidarians [44], [45]. In addition we identified nearly 500 transcripts with GO terms relating to DNA repair mechanisms including MGMT, ATR/ATM, Rad52 family proteins, XP family genes, TFIIH and RPA complex members, as well as numerous transcription factors, polymerases and ligases involved in the various DNA repair pathways (Figs 3 & 4).

#### p53 Homologs

p53 proteins play a central role in the response to DNA damage in vertebrates by initiating pathways leading to apoptosis, or cell cycle arrest and DNA repair [46]. Canonical p53 is restricted to vertebrates, but ancestral homologs (p63/73) are found throughout the metazoans (Fig. 5), and some of the DNA repair related functions of p53 are also associated with p63 [46], [47]. Surveys of the *N. vectensis* genome reveal three p53 family members (NVp63, pVS53a, and pEC53) [48] and homologs of all three variants were identified in *A. palmata*.

Divergence time between the Actinaria (anemones) and the Scleractinia (hard corals) is approximately 225 mya and divergence between the Cnidaria and Bilateria is estimated at 650–1000 mya [49]. During this time p53 family proteins have been duplicated and diversified yet their role in protecting the cell from genomic instability has been maintained [50]. p63's role in mediating apoptosis in germ line cells following DNA damage highlights its importance in mitigating genotoxic stress (Fig. 8) [48]. This function is important in long lived organisms exposed to high levels of UV radiation. Both *N. vectensis* and *A. palmata* are potentially very long lived, as genets can reproduce asexually more or less indefinitely. Thus, minimizing the impact of DNA damage to somatic and germ line cells is critical to survival and fitness. Indeed, our analysis suggests that p53 family proteins have experienced positive selection along the lineage separating Cnidaria from Bilateria (Fig. 5; Information S2). Additionally, the majority of genes involved in the canonical p53 mediated stress response (as characterized in model organisms) were present in *A. palmata* (Fig. 4).

### Paralog Group Analysis

Paralog group analyses have proven informative in identifying gene and genome duplication events and how they relate to speciation in a variety of organisms [51], [52]. We employed a conservative reciprocal best hit approach [53] to screen for duplicated genes in the *A. palmata* transcriptome. We found similar patterns in the group size distributions of paralogs in both the *N. vectensis* genome and the *A. palmata* transcriptome. While it is possible that additional paralogous copies of some genes were not sequenced, such missing transcripts add additional conservatism to this analysis.

Given the rarity of large paralog groups, members of such groups must serve important functions to justify the maintenance of multiple copies. The largest paralog group in *A. palmata* consisted of immunoglobulin superfamily proteins in the IgLON family involved in the organization of neuronal connections in the developing nervous system in other metazoans [54], [55]. Interestingly, four paralogs of another group of Ig superfamily genes involved in nervous system development, the NCAMs (neural cell adhesion molecule like genes), have been identified in *N. vectensis* and are expressed in developing larvae and planulae [56]. Further investigation of these gene families in cnidarians is of interest because of their role in the developing nervous system [57].

The second most abundant paralog group consisted of homologs of the TRAF proteins. Several TRAF homologs showed multiple paralogs in *A. palmata*, including TRAF 2, 3, 6 and a lipopolysaccharide induced TNF alpha factor. In mammals TRAF proteins are important signal transducers mediating innate immune response, apoptosis, bone metabolism and response to stress (including DNA damage) [58], [59].

These annotations were determined solely by bioinformatic means, thus further verification is required, but it is of interest to note that the six TRAF proteins found in vertebrates are thought to be the result of an evolutionarily recent diversification because few TRAF homologs have been detected in *Drosophila* and *Caenorhabditis* (n = 3 & 1 respectively) [60]. Thus the presence of multiple paralogs of the TRAF proteins in cnidarians may indicate more ancestral diversity in this protein family than previously acknowledged.

### Genetic resources for *A. palmata*

#### Microsatellite Frequency Distribution

Microsatellite repeats commonly occur in portions of transcribed mRNA including 5′ and 3′ untranslated regions [61], [62]. In the *A. palmata* transcriptome numerous microsatellite repeats were observed (n = 333), with tri and hexanucleotide motifs representing the most common types (Fig. 7A). This is true in coding sequences of many organisms [63], [64], [65] including *A. millepora* (pers. obs.) [66], because mutations in other size classes can result in frame shifts.

Among trinucleotide repeats, motifs with high AT content were most frequent in both acroporid transcriptomes (Fig. 7B). Specifically, ATC motifs were most numerous, followed by AAN type motifs. This is surprising because variants of some of these high frequency motif classes (ATC and AAT) can code for stop codons. There was a complete lack of CG and CCG variants and trinucleotides containing more than one G or C were low (<10%). This contrasts with findings from crop plants and vertebrates where motifs with higher GC content were frequent, but agrees with findings from other land plants, and fungi [63], [64], [65].

#### SNP Characterization

The frequency of SNPs in *A. palmata* was 1 per 272 bp (n = 33,433 SNPs in 14,613 contigs). The frequency in *A. millepora* was slightly higher (1 per 207 bp) [9] despite the greater depth of coverage and broader geographical origins of the *A. palmata* samples. Only a small set of SNPs were chosen for preliminary validation here, but their wide distribution and the presence of frequency differences in even a small sample size is encouraging (Information S4). Upon further validation and marker development, these SNPs will become a vital resource for studies of population structure and adaptive variation in *A. palmata* [67], [68].

#### Transition Transversion Ratios

The transition bias is a phenomenon observed in vertebrates and invertebrates where despite a twofold higher probability of occurrence, transversions are far less frequent than transitions. The distribution of SNPs in *A. palmata* and *A. millepora* species was similar in terms of the proportion of transitions to transversions with some variation in the proportion of transition and transversion types (Fig. 8). While Ts/Tv ratios around 2 are considered common, this has only been evaluated in a small number of model organisms [69], [70]. Recent findings suggest that some non-model species may not display such transition biases [71]. The transcriptome wide Ts/Tv ratio of 2.4 in *A. palmata* suggests that the transition bias does exist in this species.

Transversions are more likely to be removed by selection because there is a greater probability that they will result in an amino acid altering change. Thus deviations from a ratio of two may act as a rough test for the presence of selection [72], [73]. Transcripts with the highest 5% of ratios (Ts/Tv>5), a potential indicator of positive selection, were enriched for GO terms associated with nucleic acid metabolism and pigmentation. Transcripts with the lowest 5% of ratios, a potential indicator of purifying selection, were enriched for carbohydrate and protein metabolism functions as well as the formation of structural molecules (Table 3; Information S3). While this is only a rough test, these transcripts may serve as a useful starting point for investigations of targets of selection in corals. Indeed, the processes highlighted here are of primary importance when considering the unique metabolic adaptations of corals to their dinoflagellate symbionts. Specifically, the coral host metabolizes not only the life sustaining carbohydrates, but also the damaging oxygen radicals that are released by the photosynthesizing algae [74]. These processes occur in conjunction with exposure to high light and UV levels [75] necessitating a simultaneous response to light and heat stress which likely involves pigmentation molecules and protein chaperones in addition to a suite of metabolic and regulatory genes.

### Conclusion

Our analyses highlighted many processes conserved between corals and more complex bilaterian animals that contribute to corals' ability to maintain genomic integrity despite exposure to high levels of UV, ROS and prolonged life spans. Enrichment of these functional categories in the *A. palmata* transcriptome underscores their importance to coral survival and fitness.

This dataset provides a comprehensive look at the genes expressed in *A. palmata*. The resources developed from this dataset provide tools for coral researchers and will help generate insights into functional diversity within and among wild populations. More importantly perhaps, this sequence data enables scans for selection to identify functional variation in coral genes that contribute to their ability to adapt to changing environmental conditions.

## Methods

Collection and CITES export permits (where applicable) were obtained from the local authorities (Puerto Rico Department of Natural and Environmental Resources, the Florida Keys National Marine Sanctuary, the Florida Fish and Wildlife Commission, Caribbean Research & Management of Biodiversity in Curacao) for all samples used in this study.

### Larval Experimental Treatments and Sampling

*Acropora palmata* adults contain intracellular symbionts known as zooxanthellae. Like many other corals, symbionts are taken up after settlement and metamorphosis of the planula larvae [26]. To enrich for host genetic material and avoid a large contribution of symbiont transcripts larval tissues were targeted for RNA extraction.

To obtain larval tissue, gametes were collected from adult colonies as they were released during annual spawning events in 2008. Larvae were acquired from colonies at multiple locations in the upper Florida Keys, Puerto Rico, and Curacao to minimize ascertainment bias [76], [77]. To generate batch crosses, gametes from multiple genets (as determined by microsatellite genotyping following Baums *et al.* [23]) were combined and incubated for one hour to ensure fertilization then rinsed in filtered sea water. The resulting embryos were incubated in aquaria simulating environmental stress conditions including three temperatures and elevated CO_2_ levels.

Low, mean, and elevated temperatures (25, 27, and 30°C respectively) were used to stimulate the expression of thermal stress response genes. Larval batches were housed in 1 L plastic containers with mesh sides, suspended in four separate 45 L plastic bins filled with filtered sea water at each treatment temperature. Water was circulated with an aquarium pump and changed daily with filtered sea water preheated to the target temperature. Temperatures were maintained within ±1°C with aquarium heaters and chillers, and were monitored with HOBO data loggers (Onset Computer Corp., MA).

Larval samples were collected throughout development, from immediately following fertilization until day five, to include a full range of developmental genes in the sequencing results. Approximately 100 larvae from each container at each time-point were incubated in RNA later (Ambion, TX) then frozen in liquid nitrogen following manufacturer's recommendations. Samples were stored at −80°C prior to extraction. Additional samples from adult colonies were also collected to include a component of post metamorphosis transcripts.

For the CO_2_ treatments, larvae from Florida were raised in the Climate Change Laboratory at the Rosenstiel School of Marine and Atmospheric Science, University of Miami, Miami, FL. Water in the treatment aquaria was held at 28°C and CO_2_ concentration was manipulated by bubbling with CO_2_-enriched air to maintain control and high CO_2_ treatment conditions (400 and 800 µatm respectively) as described in Albright et al. [78]. Larvae were collected at 12 hour intervals from 36 to 84 hours of development.

### RNA extraction and Sequencing

Purification of RNA from all samples was performed using a modified Trizol extraction protocol. Samples were removed from the preservative and immediately submerged in 1 ml of Trizol for 5 minutes. Following the addition of 0.2 ml of Chloroform and incubation for 3 minutes, the samples were centrifuged at 12,000 rpm for 15 minutes at 4°C to separate the phases. The upper aqueous phase was collected and mixed with an equal volume of 70% ethanol, then applied to a Qiagen RNeasy mini spin column (Qiagen, CA) and purified following manufacturer's instructions. Sample concentration was determined using a Nanodrop ND-1000 spectrophotometer (Thermo Scientific, FL) and RNA quality was assessed with an Agilent Bioanalyzer (Agilent Technologies, CA).

A total of 71 samples, including 1 to 5 replicates each of 13 larval developmental time-points from all 3 locations, 10 CO_2_ treatment samples, and 4 adult samples from Puerto Rico and Curacao were pooled into a single batch sample such that the contribution of genetic material from each geographic location was equivalent (∼17 ug each). An aliquot of the resulting pool was submitted to the Indiana University Center for Genomics and Bioinformatics (Bloomington, IN) for cDNA preparation, normalization and sequencing on the 454-GS FLX using Titanium chemistry following previously published methods [9], [79].

### Assembly and Annotation

The resulting sequencing reads were preprocessed and annotated using a custom perl pipeline (pipemeta) following the methods of Vera *et al.* [10]. Briefly, 454 sequences were quality trimmed to filter out reads of dubious quality (>1 N, average quality <20, length <280 or >530) and screened for primer sequence using the smartscreener script in the pipemeta package (SMART “CAP” primer 5′-AAGCAGTGGTATCAACGCAGAGT-3′), then entered into seqman pro v8 (DNAstar) for additional quality filtering and assembly. Default quality filtering parameters for 454 data were used, and assembly was performed in seqman using parameters suggested by the manufacturer for short read data. Prior to assembly an additional 36,239 high quality EST Sanger reads from a pool of *A. palmata* samples from different life stages and stress conditions were included to promote the assembly of long contigs. Sanger sequences are available from the SymBioSys database (sequoia.ucmerced.edu/SymBioSys/). 454 sequences and quality scores generated in this study are available from the NCBI short read archive (Accession #: SRP006958; http://www.ncbi.nlm.nih.gov/sra), and the assembled annotated sequences can be downloaded from http://main.g2.bx.psu.edu/u/nickpolato/h/apalmataassembly.

The resulting contigs and remaining singletons were aligned with blastx to the complete Uniprot database (release 2010_05; www.uniprot.org/downloads). Acceptable hits were determined by a bitscore of >45 and a corresponding e-value of <1^−5^. Because two of the most closely related genomes in publicly available databases (*Nematostella vectensis* and *Branchiostoma floridae*) are largely unannotated, a large proportion of the highest ranking blast hits were uninformative. To maximize the information content, results were augmented with lower ranking (but more informative) annotations using a perl script and a list of keywords to avoid (i.e. ‘uncharacterized protein’ and ‘predicted protein’). Contig and singleton sequences were also submitted to the KEGG automated annotation server (www.genome.ad.jp/tools/kaas/) for further functional annotation.

### Transcriptome Completeness

A blast search was performed comparing all *A. palmata* transcriptome data against a subset of transcripts from the *N. vectensis* genome project (“*transcripts.Nemve1FilteredModels1*” downloaded from genome.jgi-psf.org). The *N. vectensis* sequences were first compared to themselves to eliminate multiple copies of sequences with >90% similarity (i.e. multi copy genes). Only blast hits between *A. palmata* and *N. vectensis* sequences with a bitscore ≥45 were considered. The length of the *A. palmata* sequence was then divided by the length of the *N. vectensis* transcript to obtain a percent coverage estimate for each unigene (Information S1), using only the best unique hit for each query and subject. This method provides a reasonable estimate of coverage because if only partial transcript sequences were obtained for the majority of *A. palmata* genes one would expect an abundance of low length ratios. Additionally, a set of 119 orthologs conserved across the metazoans and single copy in the cnidarians (*Hydra magnipapillata* and *N. vectensis*) was downloaded from OrthoDB (cegg.unige.ch/orthodb4) [80]. blast was used to identify homologs of these genes in the *A. palmata* data.

### Functional Analysis

Pathway analysis was performed in the Ingenuity Pathway Analysis (IPA) software. IPA is a web-based application that performs functional enrichment analyses to determine the probability that a given gene set is associated with pre-defined reference pathways beyond what would be expected by random chance. Pathway data in IPA is based on information in the Ingenuity Knowledge Base which is a manually reviewed database of pathways and relationships taken directly from the primary literature and public sources including GO, KEGG and EntrezGene. While this software is capable of other functions that consider gene expression levels based on microarray or RNAseq data, our analyses used only presence or absence of pathway components in the *A. palmata* dataset to compare with functional pathway relationships known in other model organisms. IPA was then used to test whether some pathways were more highly represented then others considering the number of sequences from *A. palmata* that mapped to a given pathway and the pathways' size. This application of IPA did not depend on expression level (i.e. the number of sequencing reads observed per gene). This is appropriate here because the *A. palmata* transcriptome was generated from a normalized library. IPA bases its statistical analyses on a Fisher's Exact Test (corrected for multiple testing) which is comparable to other well-known enrichment analysis methods [81]. However experimental evidence has shown that it performs better on large datasets than other similar methods [82].

A canonical pathway analysis identified those pathways in the Ingenuity Knowledge Base that were the most well represented in the *A. palmata* dataset. Our analyses compared successfully mapped *A. palmata* transcripts to the IPA reference set consisting of all molecules present in the Ingenuity Knowledge Base. Significance of the association between the *A. palmata* data set and a reference pathway was measured in two ways: 1) A ratio was calculated by dividing the number of *A. palmata* transcripts that map to a given reference pathway by the total number of molecules associated with that pathway in the Ingenuity Knowledge Base, and 2) A Fisher's exact test determined the probability that there was a significant enrichment of *A. palmata* transcripts mapping to a given pathway beyond what was expected by chance alone given the total number of genes involved in that reference pathway. Because some pathways are better characterized than others these results should be interpreted as a guide for selecting conserved and well annotated functional metazoan pathways present in the *A. palmata* transcriptome.

#### Testing for Natural Selection in p53 family genes

Tests of natural selection in p53 family genes were performed with the codeml program in paml [38]. 498 bp of the conserved DNA binding domain from two *A. palmata* homologs of *N. vectensis* genes (pVS53a and p63) were aligned with 17 other sequences representing a variety of taxa from anemones to humans (see Information S2 for gen bank accession numbers) using the global homology strategy in mafft (mafft.cbrc.jp/alignment/server/index.html). jmodeltest [83] was used to determine the appropriate substitution matrix and a maximum likelihood phylogenetic tree was generated using garli (www.molecularevolution.org/software/phylogenetics/garli) with the TIM2+G model. One hundred bootstrap replicates were performed and bootstrap values were matched to the maximum likelihood tree using sumtrees [84]. The branch-site model in paml [85] was used to test for evidence of selection along the branch separating cnidarian and bilaterian sequences. Tests considered different dN/dS ratios (i.e. the ratio of non-synonymous substitutions per non-synonymous site (dN) to synonymous substitutions per synonymous site (dS)) across the phylogenetic tree. A likelihood ratio test (2ΔlnL = 2(lnL_h0_−lnL_h1_)) of the difference between the log likelihood of the null model (lnL_h0_; NSsites = 1a) relative to that of the alternative model (lnL_h1_; NSsites = 2a), that incorporated positive selection along the branch leading to the Cnidaria by allowing the dN/dS ratio to be >1 along that branch, was performed and the result compared to a χ^2^ distribution to assess statistical significance.

### Paralog Analysis

The identification of in-paralog groups in *A. palmata* and *N. vectensis* was assessed using inparanoid v4.1 [53]. A set of 27,273 filtered protein models from *N. vectensis* (downloaded from genome.jgi-psf.org), and 43,081 ORFs from the *A. palmata* transcriptome were used in the analysis. Acceptable hits were determined by a bitscore of >40 using the BLOSUM62 scoring matrix.

### Development of Genetic Resources

Potential SNPs were detected in contigs with sufficient depth of coverage using the snphunter script in pipemeta [10]. The resulting annotation and SNP data were archived in a searchable mysql database and will be made available to the public via the Dryad data archive (http://datadryad.org/) along with sequences containing potential microsatellite markers.

To confirm the results obtained by snphunter SNPs were validated using PCR and the amplified products were sequenced on an ABI3700. Primers were designed using primer3 (frodo.wi.mit.edu/primer3/; Information S4) to amplify portions of six contigs containing putative SNPs in a sample population which consisted of 11 individuals from Florida, 8 from Curacao, and 7 from Puerto Rico. Equal volumes (0.2 µl) of forward and reverse primers (5 uM each) were combined with 20–200 ng of template DNA, 1× NH_4_ Reaction Buffer (Bioline, MA), 2 mM of MgCl_2_, 0.2 mM of dNTPs, and 2 U of Biolase polymerase (Bioline, MA). PCR was carried out in an Eppendorf Mastercyler Gradient with an initial denaturation step of 95°C for 5 min followed by 35 cycles of 95°C for 20 s; annealing at 56°C for 20 s; and 72°C for 30 s, and a final extension of 30 min at 72°C.

Transition and transversion ratios were determined based on the number of SNPs in each class identified by snphunter for each contig. Enrichment analysis of GO terms associated with transcripts showing the top and bottom 5% of all Ts/Tv ratios (<1 or >5) was carried out using wego (http://wego.genomics.org.cn/cgi-bin/wego/index.pl) [86].

To detect repeat elements, the full set of sequences was uploaded to the Tandem Repeats Database (http://tandem.bu.edu/cgi-bin/trdb/trdb.exe) [87] and searched using tandem repeats finder v4.04 [88]. Repeats between 2 and 6 bp were considered and comparisons were made with microsatellite frequencies from the publicly available *A. millepora* transcriptome (www.bio.utexas.edu/research/matz_lab/matzlab/Data.html) [9].

## Supporting Information

Information S1 **Distributions of log length ratios for** ***A. palmata*** **and** ***N. vectensis*** **transcripts, and fragmentation levels as compared to a set of 119 single copy orthologs.**
(XLSX)

Information S2 **Results of tests for natural selection using the program codeml.**
(XLSX)

Information S3 **Top and bottom 5% of Ts/Tv ratios.**
(XLS)

Information S4 **SNP validation summary and primer sequences.**
(XLSX)
